# Supplementary material for: Are carbon emissions trading and green financial instruments synergistic? -Comprehensive quantitative research based on content analysis
Source: PLoS One. 2024 Mar 7;19(3):e0298601. doi: 10.1371/journal.pone.0298601 (PMC10919601; doi:10.1371/journal.pone.0298601)
Supplement: S1 Data — (ZIP) [file pone.0298601.s001.zip › Data/地级市Z_F工作报告.zip!/╔┬╬≈/╙▄┴╓╩╨/└┤╫╘╣1⁄2╓┌║┼í╛┬φ┐╦╩2╛▌═°í┐_2015.docx]

各位代表：

现在，我代表市人民政府向大会作工作报告，请予审议，并请市政协委员和其他列席人员提出意见。

一、2014年工作回顾

刚刚过去的2014年是我市发展极为艰难的一年，也是我们迎难而上、务实奋进，取得发展新成就的一年。一年来，面对经济下行、能化产品价格下跌、民营经济运行困难、各类风险不断加大的严峻形势，我们牢牢把握稳中求进工作总基调，认真贯彻中省和市委的决策部署，统筹推进稳增长、促改革、调结构、惠民生、防风险各项工作，经济发展稳中有进、稳中向好。全市生产总值突破三千亿大关，达到3005.7亿元，增长9%；完成全社会固定资产投资1647亿元；财政总收入677.6亿元，增长5.2%，其中地方财政收入267.8亿元，增长13.3%；城乡居民收入分别达到29665元、9730元，增长10.6%和12%。

特别需要给各位代表报告的是，在极其困难的情况下，我们取得了几项令人振奋的新成绩。一是转型升级取得重大突破，陕西有色铝镁合金、靖边能化园区一期、中煤榆横煤制烯烃等4个超百亿的骨干项目和延长煤油共炼等一批资源深度转化项目建成投产，新增工业增加值176亿元，有力地支撑全年经济增长。二是煤炭产业实现产销两旺，抓住国家治污降霾政策机遇，强力推进“榆林煤”“榆林兰炭”北上京津冀、东进长三角、南下川湘渝，走活了一盘棋，全年生产原煤3.63亿吨，销售3.87亿吨，产销率达到106.7%，创历史最好水平。三是兑现了中心城区“一年打基础、两年见成效、三年大变样”的承诺，实现了建成区面积、道路里程、绿化面积、学校学位数量、医院新增床位“五个翻番”，城市功能和品位明显提升。四是“三年植绿大行动”圆满收官，累计完成投资65亿元，造林303万亩，全市860万亩流沙全部得到治理，森林覆盖率提升2.3个百分点，达到33%，生态建设成效显著。

一年来，我们紧扣目标、攻坚克难、埋头苦干，主要抓了以下工作：

（一）加强调控，经济保持平稳较快增长。把稳定工业经济增长摆在更加突出的位置，及时跟进中省政策，出台工业稳增长十条意见，加强对重点行业、重点企业调控和扶持，促进产能释放和产销衔接，工业经济平稳运行。20种主要工业产品中18种实现正增长，产销率达到96.4%，提高2.1个百分点，实现规模以上工业增加值1974亿元，增长10%，总量连续七年位居全省第一。积极发挥投资的关键作用，进一步夯实“五个一”工作责任，创新“一企一策”工作模式，强力推进重大项目建设，年初安排的105个重点项目完成投资456亿元。全力拓展消费的基础作用，加强物价调控，培育电子商务、金融物流等消费新热点，促进城乡消费较快增长，实现社会消费品零售总额321亿元，增长7.6%。

（二）主动转型，产业结构调整步伐加快。把推进能化产业转型升级作为调结构的主攻方向，强力推进骨干项目建设和关键技术研发，靖边能化园区全球首套煤油气综合转化装置一次试车成功，粉煤中低温热解等技术攻关取得新进展，兰炭在高炉喷吹、民用清洁燃料等领域应用取得新突破。大力发展战略性新兴产业，全市风电、光电并网装机容量达到158万千瓦，百万千瓦风电基地全面建成，我市入选国家第一批新能源示范创建市。不断加大科技创新工作力度，成立全省第一个产学研合作联盟，建立企业研发中心、实验室50家；成交技术合同21件，成交额5亿元；专利授权量475件，增长31%；科技对经济增长的贡献率达到45%。积极推进各大产业园区建设，高新区GDP达300亿元，榆神工业区入围全国化工园区20强，锦界工业园成为全省唯一国家级循环经济示范园区。加快文化旅游产业发展，统万城和石峁2个遗址公园列入全省30个重大文化项目，米脂县荣获“中国千年古县”称号，佳县泥河沟千年枣园入选全球重要农业文化遗产，全年实现旅游综合收入95亿元，增长27%。

（三）强基提质，农村经济持续健康发展。把“三农”工作放在全局工作的重中之重位置，紧盯农业现代化发展方向，推动农业发展方式转变。“五个百强”建设扎实推进，全市现代农业园区数量、旱地全膜双垄沟播玉米面积、农产品质量安全实验室建设及检测能力、土地流转面积、家庭农场总数、高级职业农民认定总数6项指标位居全省第一，成为全国唯一实行产品质量安全追溯系统整市推进的地级市。新创2项旱地谷子全国高产纪录，粮食总产量达到158万吨，粮食生产实现“十一连丰”，成为全国马铃薯生产第三大市。羊子、生猪、家禽饲养量分别达到1023万只、249万头、1077万只，山羊绒产量达到2019吨，成为全国非牧区第一养羊大市和全国山羊绒产量第一大市。新建基本农田8.7万亩，新建加固维修淤地坝867座。新建供水工程788处，解决了32万人的安全饮水问题。改造县乡公路310公里，完成通村油路1500公里。扶贫开发整村推进204个，移民搬迁6264户，完工3708户，11.7万人实现脱贫。

（四）倾力扶持，民营企业发展困难逐步缓解。把加快民营经济发展作为富民强市的重要支撑，及时研究出台36条政策措施，帮助民营企业尽快走出困境。市县财政投入2亿元实施“助保贷”业务，撬动银行20亿元为中小企业贷款。组织政银企对接活动5次，促成银行与企业签订合同或意向贷款476.8亿元，落实资金441.6亿元；矿权抵押贷款政策取得突破，部分涉煤民营企业融资难问题进一步缓解。深入实施神府省级民营经济转型升级试验区十大行动计划，选聘50名优秀高校毕业生到民营企业服务，非公经济运行整体好转，民营企业家的发展信心逐步恢复。实现非公经济增加值1214亿元，增长6.9%，占全市GDP比重达40.4%。

（五）统筹发展，城乡面貌持续改善。把统筹城乡发展作为加快城镇化建设的有力抓手，持续加大中心城市、县城、重点集镇建设力度。中心城区累计开工市政项目6大类57个，完成投资13.6亿元。榆林大道北段等14个项目建成投运，长城北路等18个项目加快建设，榆溪河生态长廊、东沙生态公园、空港沙地森林公园三大城市“绿肺”建成开放，新增绿化面积180万平方米，绿化覆盖率达38.3%。成功创建省级环保模范城市、省级文明城市，全国卫生城市创建通过了考核验收。府谷率先在全省建成省级环保模范县，清涧、定边成功创建省级园林城市；锦界、东坑省级重点示范镇和“双十二”试点镇村建设扎实推进，分别完成投资5.7亿元和5.8亿元，全市城镇化率达53.9%。“四位一体”的振南帮扶机制持续发力，全年实施振南项目50个，完成投资6.5亿元，南部六县发展后劲增强。

（六）多措并举，发展保障能力得到提升。把破解瓶颈制约作为推动可持续发展的根本举措，全力拓展经济发展空间。加快基础设施建设，准神铁路建成投运，神朔铁路完成扩能改造；榆佳高速、绕城快速干道北段通车，沿黄公路一期建成通车；王圪堵水库开始供水并正式上网发电。狠抓节能减排工作，大力实施“蓝天、碧水、宁静、清洁”四大工程，建成火电脱硫脱硝设施53台，淘汰落后产能16.4万吨，万元工业增加值用水量下降5.3%，中心城区最后39台燃煤锅炉完成改造，四项主要污染物年度减排任务全部超额完成，市区、各县城污水处理率分别达到85%和75%，垃圾处理率分别达到95%和75%。启动PM2.5监测工作，全年好于二级以上天数达到336天，较上年增加14天。

（七）深化改革，发展活力不断增强。把转变政府职能作为全面深化改革的中心任务，坚持问题导向，加快推进行政、经济、农村、社会等领域改革步伐。累计承接中省取消下放行政审批事项102项，精简市级行政审批事项79项，取消带有审批和管理性质的“红头文件”87件，“营改增”试点扩大到铁路运输、电信服务等领域，农村承包地、宅基地、集体建设用地确权登记颁证工作稳步展开。加快商事制度改革，新登记市场主体3.5万户、新增注册资本金712亿元，分别增长19.4%和17.6%。启动全省首批“科技金融结合试点市”工作。在全省率先组织实施企业直供电交易，3家企业实现电力直接交易。启动运行市级污染源监控平台，排污权交易试点工作走在全省前列。全面推开国家“多规合一”试点工作。深入推进神木、府谷省直管县改革工作。进一步加大对外开放力度，招商到位资金615亿元，进出口总额达到4902万美元。

（八）关注民生，老百姓得到更多实惠。把改善保障民生作为政府工作的首要任务，按照兜底补短原则，全年投入346亿元，办好民生实事。中心城区14所新建学校和1所改扩建学校投入使用，上学难、班额大问题基本解决。市一院二期住院综合楼主体建成，市二院迁建工程完工。开发公益性岗位3200个，新增城镇就业2.8万人，转移农村劳动力66.2万人。启动城镇职工基本医疗保险市级统筹，医疗救助报销比例从60%提高到65%，城乡居民养老保险实现全覆盖；新农合筹资标准提高到480元，高出全省标准50元。新开工保障性住房3.23万套，建成1.72万套。新闻大厦主体完工，神木县艺术大厦等文化项目加快推进。实施城乡体育惠民工程164项，开展大型全民健身活动220次，我市运动员在省十五届运动会上，取得188金、82银、70铜的历史最佳成绩。

（九）创新治理，社会大局和谐稳定。把维护社会和谐稳定作为推进改革发展的基本保障，大力推进社会稳定风险评估机制，全年评估重大项目424个，从源头上预防和减少了社会矛盾的发生。强化各级领导干部接访下访，夯实信访责任，妥善处置了一批突出问题，进京赴省上访分别下降34%和41%。妥善处置民间借贷纠纷，加快非法集资案件侦办进度，民间借贷问题逐步趋于平稳。推进城区巡防机制改革，加快立体化防控体系建设，新建各类探头4万多个，全市治安、刑事发案“双下降”，社会治安满意率达91.95%，增幅全省第一。全力推进“政府监管网格化、行业直管专业化、企业管理精细化”建设，强化责任追究，安全生产形势总体平稳。扎实开展双拥模范城创建活动，拥军优属“九件实事”累计投入3000多万元，为历年之最。

（十）转变作风，行政效能明显提升。把开展教育实践活动作为加强政府自身建设的有效载体，持续发力扫“四风”、转作风、树新风，全力打造为民、务实、清廉、服务型政府。市政府班子教育实践活动整改措施全面落实，各部门及时解决了一批群众反映的突出问题。认真贯彻落实“八项规定”，“三公”经费同比下降22%，公开拍卖超标公务用车425辆，清理超面积办公用房13.7万平方米，已立项未开工的8个楼堂馆所项目全部停建。深入开展群众满意服务窗口创建活动，窗口单位普遍实行一站式办理审批和服务，市政务服务中心及榆阳、府谷、绥德等县区政务服务中心扩容投运。自觉接受人大依法监督、政协民主监督和社会舆论监督，办理人大代表议案和建议158件、政协提案392件，办复率均达100%。

各位代表，过去的一年，我们顶住了压力，扛住了风险，战胜了困难，全市经济社会保持了平稳较快发展的良好势头。这是市委统揽全局、坚强领导的结果，是市人大、市政协有效监督、鼎力支持的结果，是全市人民团结奋斗、拼搏进取的结果。在此，我代表市人民政府，向全市广大干部群众、驻榆部队指战员、武警官兵、公安干警致以崇高的敬意！向大力支持政府工作的各位代表、委员和社会各界人士表示衷心的感谢！

在总结成绩的同时，我们也清醒认识到，榆林发展仍然面临一些突出问题和深层次矛盾：产业结构不尽合理，转型升级任务艰巨；科技创新能力不强，应对市场风险的能力依然较弱；宏观经济持续下行，市场主体投资信心不足，民营企业恢复性增长的基础还不牢固；各种潜在风险因素逐渐显现，困扰发展的新老问题交织，维护稳定任务不断加大；有法不依、执法不严等现象依然存在，干部作风有待进一步改进，法治政府建设任重道远。对此，我们将在以后的工作中努力加以解决。

二、2015年工作的总体思路和预期目标

当前，宏观经济环境正在发生深刻变化。中央作出了我国经济发展进入新常态的战略判断，主要表现为速度变化、结构优化、动力转换的特征。我们要充分认识新常态，主动适应新常态，积极引领新常态，在新常态下有新作为。

新常态下挑战与机遇同在。我们必须承受过去经济发展井喷式增长到经济增速回落及波动带来的压力，必须承受资源依赖和投资拉动粗放式发展面临的结构调整压力，必须承受多年来经济高速发展积累的一些隐性风险显性化带来的压力。但新常态也孕育着新的发展机遇。从长远来看，榆林经济稳健发展的基本面没有改变，国家能源化工基地的定位没有改变，榆林作为陕西经济增长重要一极的地位没有改变。我们有信心变压力为动力、化挑战为机遇，在新常态下赢得新发展。信心来源于，榆林的发展已经上升到国家战略层面。我市纳入呼包银榆经济区、呼包鄂榆城市群、振兴陕甘宁革命老区、国家能源战略行动计划（2014—2020年）等一系列国家级规划，随着各项规划逐步落实，我们将获得更多的政策支持。信心来源于，省委、省政府对榆林发展的高度重视。定位榆林为全省经济增长的重要一极，先后出台新“27条”、神府省直管县、促进神府民营经济转型升级等一系列政策措施，为我市加快发展注入了活力。信心来源于，我们按照“三个转化”要求推进能化项目建设打下的坚实基础。“十二五”以来，我们累计投入近3000亿元，实施了7个过百亿、5个50—100亿、19个10—50亿元等一批投资规模大、科技含量高、带动能力强的资源深度转化项目，一批新的增长点正在加速形成，榆林经济在新常态下仍然会大有作为。

做好新常态下的各项工作，我们将更加注重转变观念，打破一味追求高速增长的惯性思维，把提高增长的质量和效益放在首位，把科技创新作为加快发展的主动力，把转型升级作为结构调整的主抓手，全面推进榆林经济迈向中高端。更加注重扬长补短，继续挖掘潜力，将各方面的比较优势尽快转化为经济成效；坚持问题导向，着力破解发展中不平衡、不协调、不可持续的问题，推进经济社会全面协调可持续发展。更加注重统筹衔接，以“建功必须有我”的责任担当、“功成不必在我”的开阔胸襟，既脚踏实地做好当前各项工作，又立足长远谋划未来发展，打好“十二五”的收官战，谋好“十三五”的开局篇。

综合研判形势，2015年政府工作的总体思路是：全面贯彻落实党的十八大、十八届三中、四中全会和习近平总书记系列重要讲话精神，坚持稳中求进工作总基调，以“一中心两基地”建设为统领，以提高经济发展质量和效益为中心，狠抓改革攻坚，突出创新驱动，加快产业转型，加强民生保障，推进法治建设，强化风险防控，促进经济平稳健康发展和社会和谐稳定。主要预期目标是：全市生产总值确保增长9%，力争达到10%；全社会固定资产投资增长11%左右；地方财政收入同口径增长12%；城乡居民人均可支配收入增长10%；居民消费品价格涨幅控制在3.5%以内；人口自然增长率控制在6.5‰以内；城镇登记失业率控制在3.9%以内。

三、2015年主要工作任务

（一）全力推动经济平稳较快增长。发展是硬道理，发展是解决榆林一切矛盾和问题的关键。必须抓紧经济建设中心不放松，扭住发展第一要务不动摇，敢于担当、奋发有为、务实苦干，不断巩固陕西经济重要一极的地位。

稳定工业增长速度。坚持工业的主体地位不动摇，进一步强化分析研判和运行调度工作，健全完善各类支持政策举措，促进产销衔接和产能释放。积极支持陕西有色铝镁合金、中煤榆横烯烃等重大能化项目正常生产；确保未来煤制油、神华甲醇下游加工等项目投产达效，争取大海则、巴拉素等7个煤矿取得核准，全年新增工业增加值180亿元。继续加大煤炭促销力度，加快煤炭外运绿色通道建设，积极开拓华北、华东、西南、关中等地市场，让“榆林煤”“榆林兰炭”净化京津冀和关中地区的空气。坚定不移地推进品牌化战略，把金属镁、聚氯乙烯等主要工业产品纳入推销范围，让“榆林好产品”畅销全国、走向世界。大力发展洗选煤产业，加大技术改造，加快重组整合，培育发展一批大型洗选煤加工项目。全年规模以上工业增加值增长10%左右。

狠抓重点项目建设。始终把项目建设作为稳增长的重要抓手，进一步强化“五个一”工作机制和“四位一体”包抓体系，切实解决各类“卡脖子”问题，扎实推进全年安排的100个重点项目建设，完成年度投资460亿元。重点加快建设西湾露天煤矿等53个续建项目，开工建设神华煤炭综合利用等47个新建项目，力争朱家峁煤矿等20个项目建成投产。加强与驻榆企业协调对接，力促中煤榆横煤制烯烃、未来煤制油等项目二期工程上马。认真研究国家支持西部发展政策，精心谋划一批能够挤进中省盘子的重大项目，积极争取各类专项资金支持。

强化基础保障能力。持续加大补短板、破瓶颈工作力度，为榆林经济长远发展增添后劲。完成神佳米（神佳段）高速、绕城快速干道西段主体、沿黄公路二期工程，启动定边至吴起高速公路、204省道靖边段改建及绥德、清涧、米脂过境线等8个项目，加快大柳塔至石马川一级公路等8个续建项目建设。加大小纪汉煤矿铁路专用线等5个项目推进力度，启动蒙西至华中、神木至靖边、神木至瓦塘等6个铁路项目建设，力争通过5年时间，把我市货运方式转变为铁路运输为主。开工建设榆佳工业园区和清涧延安延川支线供水工程，加快万镇引水工程建设，建成吴堡县城及工业园区供水一期主体工程；改造升级黄河西线供水工程，认真做好黄河东线多点引水工程前期工作，争取将碛口水利枢纽工程纳入国家“十三五”规划。积极打造神府千万千瓦级煤电一体化基地，开工建设榆横至山东输变电榆林段工程。积极推进长庆延长置换原油第二条百万吨输油管线建设。加强园区基础设施等配套保障，创新园区服务管理体制，不断提升园区的承载能力和吸引力。

扶持非公经济发展。全面贯彻落实中省市各项扶持政策，帮助民营企业尽快走出困境、转型发展。探索推广政府和社会资本合作（PPP）模式，引导民营企业进入重大能化、基础设施和社会事业领域。加大金融服务创新力度，加快推进神府民营经济转型升级试验区建设，积极推动北元化工、康隆能源等企业上市融资。鼓励支持民企、国企深度合作，实现混合所有制经济发展新突破。进一步推进矿权抵押贷款，适度增加“助保贷”规模，扩大财政风险补偿金额度，加强政银企对接，努力化解融资难问题。继续推行市级领导及相关部门联系非公有制企业制度，帮助企业解决实际困难。全年新增中小工业企业70户，非公经济增加值占全市GDP比重达到41%。

（二）全面打造榆林经济升级版。围绕特色优势产业和“三个转化”要求，继续以超百亿元重大资源转化项目为抓手，以推动服务业转型升级为基础，以提高科技创新支撑作用为核心，积极谋划实施榆林经济“三年大转型大调整”行动计划，不断提高经济发展的质量和效益。

推进工业经济转型升级。牢牢抓住当前倒逼转型的有利时机，做大做强优势产业，延伸产业链，提高附加值，增强竞争力。积极支持煤制芳烃、甲醇制烯烃、煤制油和煤制气等产业发展，开工建设华电100万吨煤制芳烃等4个煤炭深加工项目。继续推进兰炭产业转型升级，支持干馏煤气除尘、焦炉煤气提纯和煤焦油精细化加工等关键技术攻关，推进粉煤干馏工业化试验示范，鼓励定边等县区发展油气转化项目。大力发展新能源产业，年内建成府谷田家寨风电场、榆神协合生态光伏发电等一批新能源项目，新增电力装机50万千瓦。加快推进LNG加气站网点建设，支持陕汽榆林东方新能源专用车扩大生产规模、启动二期项目；推进江苏海陆重工装备制造、蓝天德汽车轮毂等项目建设，加快装备制造业发展。

加快现代服务业发展。坚持将增加群众收入和培育新型消费业态作为扩大消费的着力点，大力发展与能化基地配套的城市经济和工业物流经济。认真落实城乡居民收入与经济增长、财政收入增长联动机制，积极引导休闲、健康、养老、信用等消费新业态快速发展，推动群众消费结构升级。加快推进电子商务公共信息服务平台建设，促进电子商务和信息消费大发展。稳步推进榆横、神府、定靖、绥米四大物流园区建设，构建晋陕蒙宁毗邻地区物流枢纽。深入挖掘榆林多元文化，发展壮大文化旅游产业，积极开展统万城遗址世界文化遗产申报和国家遗址公园建设工作，做好石峁遗址发掘和保护，加大白云山、红碱淖5A景区建设力度，统一规划建设王圪堵水利风景区，支持有关县区做好长城遗址、吴堡石城、波罗古堡、龙洲丹霞地貌等保护和开发工作，努力将潜在的旅游资源优势转化为现实的经济发展优势。严厉打击制假售假、商业欺诈等违法行为，努力保持价格总水平基本稳定，为群众安全消费、放心消费营造良好环境。

全面推进科技创新。紧紧围绕结构调整方向，坚定不移地实施创新驱动战略，努力构建以企业为主体的创新体系。结合兰炭和金属镁产业转型升级的需求，加大工业科研项目实施力度，新建市级工程技术研究中心3个、重点实验室2个、产业技术创新联盟2个，着力打造榆林“清洁煤都”。充分发挥产学研联盟作用，创新科技研发、成果转化、政府服务模式，促进科研成果尽快就地转化。与西安交大、陕西煤化等单位合作组建能源研究院，为榆林能源经济发展提供科技支撑。加强与西北农林科技大学等高校的合作，进一步提升红枣、玉米、马铃薯等技术研发水平，为农业发展提供更加便捷高效的科技服务。加强信息化基础建设，探索建立电子商务园区，积极引进大数据产业发展，推动“智慧榆林”建设取得实质性进展。

（三）积极推进农业现代化。实现农业发展方式由传统农业向现代农业转变，由一家一户的分散经营向家庭农牧场的规模经营转变，由单纯的农业生产向一二三产融合发展转变，由靠天吃饭的粗放型农业向依靠科技创新的集约型农业转变，由传统农民向职业农民转变。

大力发展现代特色农业。认真贯彻中央1号文件精神，稳定粮食播种面积，培育龙头企业，支持家庭农场规模经营，加强农民职业技能培训，全年新认定省级园区5个、市级园区10个、农民专业合作社100个。紧紧抓住国家实施马铃薯主粮化战略机遇，加大对马铃薯种植、加工的政策扶持和科技支持，努力打造国家级的马铃薯生产基地。开展畜禽标准化健康养殖，突出抓好动物疫病防控和饲草加工利用，建设5个百万只养羊大县（区），羊子饲养规模达到1050万只。整合规范地方特色品牌，打造以“榆林”冠名的红枣、羊肉、小杂粮等6大地域品牌。加大农副产品流通体系建设，扶持大型农副产品流通企业发展，打造全省最大的名优农产品网络销售平台。进一步完善市县两级农产品质量安全追溯机制，加大“三品一标”认证力度，确保农产品质量安全。

不断夯实农业发展基础。大力推进农田水利基本建设，积极发展生态农业和节水农业，新建高标准农田7.5万亩，新建加固维修淤地坝850座。强化农业科技服务，加大农机具普及力度，支持靖边全国农业机械示范县创建工作；深入实施种子工程，推广测土配方施肥技术，加快推进农作物病虫害远程监控信息平台建设；强化气象服务体系建设，适时开展人工增雨、防雹作业，全面提高农业生产能力。继续实施农网升级改造工程，推进城乡供电一体化。改造县乡公路200公里，建成通村公路2000公里。巩固农村安全饮水成果，解决10万人的安全饮水问题。

扎实做好扶贫工作。认真落实中央精准扶贫、产业扶贫等相关政策，进一步扩大扶贫项目和扶贫资金的覆盖面，积极探索整合捆绑使用扶贫资金，因地制宜发展特色经济。探索设立扶贫风险补偿和担保基金制度，积极开展片区开发金融扶贫试点工作。全年实施整村推进项目204个，扶贫移民搬迁4000户1.6万人，脱贫11.7万人，全面完成2012年以来的移民搬迁任务。

（四）统筹推进城乡一体化发展。认真落实中央新型城镇化发展要求，把握“四化同步”发展规律，加快区域中心城市和新型城镇体系建设，构建城乡一体、区域协调的发展新格局。

强化中心城市建设。按照“一年补短板、两年大提升、三年变宜居”的思路，进一步完善功能、提升品位，把榆林建设成塞上宜居城市。优化完善城区路网体系，环城北路西段、长城南路延伸等20个项目建成通车。加大绿化美化力度，建成东沙生态公园二期、沙河公园等项目，开工建设榆溪河生态长廊二期和大墩梁尤家峁自然生态保护区一期工程。完善城市公共交通体系，新开通3条公交线路，新建13个公共停车场，启动城市公共自行车系统建设。加大城市地质灾害隐患点的改造治理力度，加强地下管网建设，建成榆溪河污水直排等截污干管工程，开工建设榆阳河部分河道整治工程，新建供热管网22.5公里。加快棚户区改造，积极推进沙河口、杏墕村等城中村改造项目，精心谋划一批高质量的棚户区改造项目。积极探索城市科学化、集约化、精细化管理模式，集中开展违章建筑、违章停车、店外经营、环境卫生等城市“顽疾”的综合整治，广泛开展国家环保模范城市、文明城市、卫生城市、园林城市、节水型城市、宜居城市创建活动，努力形成便捷畅通、文明有序、生态良好、宜居宜业的城市新风貌。

提升城镇承载能力。依托工业集中区和现代农业园区发展县域经济，推动神木、府谷城镇带建设，加快靖边、神木撤县设市工作。继续完善县城基础设施和公共服务设施，全面推进县城人防建设，进一步提升县城管理水平。加大重点镇建设力度，抓好锦界、东坑等9个国家级重点镇和王家砭、神木镇2个国家发展改革试点镇建设。扎实推进“双十二”试点工程，全面完成三年建设目标任务。完善城镇化配套政策，放开户籍限制，推进有条件农民进城落户，全市城镇化率达到55%。

促进区域协调发展。进一步强化“四位一体”的振南机制，加大市级支持力度，落实好北部县区对口帮扶南部县政策。结合吕梁山片区扶贫攻坚规划的实施，积极争取中省资金及项目支持，谋划实施一批带动性强、辐射面广的大项目、好项目，不断增强南部县自我发展能力。大力推进榆横一体化，加快横山撤县设区工作，实现西南新区与中心城区互联互通。

（五）打好全面深化改革攻坚战。把全面深化改革作为推动经济社会转型发展的关键一招，在不同领域进行大胆探索，逐步健全符合科学发展要求、运转高效灵活的体制机制，不断激发转型发展的内生活力和动力。

扎实推进经济体制改革。充分发挥市场在资源配置中的决定性作用，从制约发展最突出的症结改起，从人民群众最期盼解决的问题改起，加快完善现代市场体系。认真落实中省各类定向减税政策，实施好煤炭资源税从价计征改革，切实减轻企业负担。积极推进反映资源稀缺程度的阶梯水价改革。全面贯彻中省深化财税体制改革方案，进一步扩大“营改增”范围，健全政府性债务管理和风险预警机制。抓好农村土地经营权确权登记颁证工作，引导承包农户依法采取转包、出租、互换、转让及入股等方式流转承包地。探索金融服务农业的新机制，积极开展“助农贷”业务试点工作，拓宽农业融资渠道。

加快推进行政体制改革。深化行政审批制度改革，继续取消和下放审批事项，对保留行政事业性收费项目及经营服务性收费项目实行目录清单管理。稳步推动卫生计生机构及直属单位整合工作，加快推进医疗服务价格调整及支付制度改革。建立县以下机关公务员职务与职级并行制度，启动机关事业单位工作人员养老保险制度改革。加快推进不动产统一登记工作，尽快完成职责整合和机构设置。继续做好神木、府谷省直管县改革工作，积极协调理顺管理体制机制。加快推进预算管理制度改革，规范预决算公开，建立健全预算绩效评价体系。全面推进商事制度改革，研究探索工商营业执照、组织机构代码证和税务登记证“三证合一”登记制度。加快企业信用平台建设，依法加强市场监管，保护好消费者的合法权益。

全力推动规划体制改革。完成国家“多规合一”试点改革任务，实现市域空间规划“一张图”全覆盖。启动国土空间综合规划编制工作，制定全市规划管理暂行办法，构建全新的规划工作管理体制和运行机制。准确把握新常态下榆林经济发展规律，科学编制“十三五”规划，为榆林经济社会发展绘好蓝图。

不断加大对外开放力度。围绕现代煤化工、新材料、装备制造、现代农业、文化旅游等重点领域，大力开展精准招商、驻点招商、联合招商、委托招商、网络招商，全年实现招商引资到位资金600亿元。积极组团参加西洽会、农高会等招商活动，精心承办好第十届煤博会，主动融入新丝路经济带。着力转变外贸增长方式，加快推进陆路口岸查验货场建设，积极申报一类航空口岸。

（六）切实加强生态环境保护。牢固树立保护生态环境就是保护生产力、改善生态环境就是发展生产力的理念，久久为功、驰而不息地抓好生态建设和环境保护工作，努力建设天蓝水绿、山川秀美的美丽榆林。

持续加大生态环境建设。围绕“城市增绿、市民添福、山区造林、农民增收”思路，启动实施“榆林生态建设绿化大行动”。重点抓好城市绿化、绿色长廊建设等九大绿化工程，完成植树造林50万亩、人工种草100万亩、道路绿化100公里，治理水土流失1100平方公里。启动榆林国家生态保护与建设示范区工作，积极开展城区拆墙透绿、破硬植绿、见缝插绿、社区绿化、庭院绿化、工矿区绿化等工作，督促资源开采企业履行绿化责任，引导社会各界积极参与生态建设，让植绿、爱绿、护绿的生态文明理念深入人心。严格封山禁牧，强化森林防火，狠抓病虫害防治，依法打击非法侵占林地行为，巩固植树造林成果。

扎实推进节能减排。认真贯彻落实新《环保法》，严格执行节能减排目标责任制、一票否决制和“环保三同时”制度。完善落后产能退出机制，加快淘汰小火电、小水泥等落后产能，推动重点行业、产业提升技术水平。加大工业园区、重点镇污水垃圾等环保基础设施建设力度，加快火电、兰炭、化工等重点行业环保设施的建设和改造。强化水质监测，做好水源地保护工作，确保饮用水水源地100%达标。深入开展“以奖促治、以创促治、以减促治”三大整治活动，加大中心城区和城乡结合部禁烧烟煤工作力度，全面实施“气化榆林”工程，下大力气抓好城乡环境卫生整治，让榆林的天更蓝、水更绿。

加强采空区治理。严格落实“先安置、后开采”政策，做好涉煤县区煤矿采空区分布勘查工作，落实相关企业的主体责任，加快推进矿区土地复垦和采空区治理，促进矿区生态环境持续好转。继续实施好红碱淖湿地保护，积极推动红碱淖申报国家级自然保护区。

（七）着力强化民生保障水平。民生连着民心，民生凝聚民力。坚持把解决民生问题放在各项工作的首位，按照兜底补短全覆盖原则，加大投入力度，强化薄弱环节，努力建设幸福榆林。

优先发展教育事业。加快榆林四中等11所学校建设，确保8所学校秋季投入使用。全面实施第二期学前教育三年行动计划，启动44个幼儿园建设项目。积极推进义务教育学校标准化建设，横山、绥德启动“双高双普”创建，米脂通过“双高双普”评估，榆阳区创建省级“教育强区”。积极支持榆林学院升格大学申报工作，大力发展职业技术教育，加快实施特殊教育提升计划。加强师资队伍建设，严格落实校长、教师轮岗制度，实施阳光招生、均衡编班制度，集中开展乱补课、乱收费、乱推销整治活动，促进教育事业公平发展、均衡发展、健康发展。

千方百计扩大就业。督促在榆企业严格落实当地用工有关政策，继续实施大学生就业促进和创业引领计划，促进高校毕业生就业创业比例双提高。加强就业信息服务，鼓励社会资本建设创业孵化基地，为各类创业者提供平台。加大就业援助帮扶力度，强化大学生见习和公益性岗位等托底安置，解决好城镇“零就业”家庭、退役军人等困难人员的就业问题。全年新增城镇就业2.8万人，下岗失业人员再就业7000人，转移农村劳动力66万人。

完善社会保障体系。全力推进养老、医疗、工伤、生育、失业保险提标扩面工作。把大病保险纳入商业保险领域，为大病患者提供多层次、广覆盖的医疗保障；新农合筹资标准由480元提高到500元，提高的20元由市县财政承担。全面推进保障性住房建设，加快廉租房与公租房、经适房与限价房并轨工作，探索推行保障性住房社会化收储新模式。加快以社区为依托的“居家养老”试点工作，完善农村留守儿童、妇女、老年人、残疾人关爱服务体系。继续扩大社会救助范围，更加关注低收入群众生活，实现特困人群救助全覆盖。

加快发展医疗卫生事业。完善医疗服务体系，完成市一院绥德院区改扩建、榆林院区二期新建和市二院新址整体搬迁工程，加快市三院建设进度。大力开展人才培养和重点专科建设，提升各级医疗机构服务能力和水平，每个县区建成一所二级甲等综合医院。启动居民健康一卡通工程，实现基本公共卫生信息化管理。认真落实有关政策，积极支持中医发展。创新人口服务与管理机制，促进全市人口健康均衡发展。

实施文体惠民工程。加大文化事业投入力度，建立市县政府购买公共文化服务长效机制。加快推进榆林文化艺术中心等文化项目建设，启动市体育中心项目建设，促进竞技体育与群众体育全面发展。加大乡村集中居住区文体设施建设力度，继续实施地方节目无线覆盖工程，全面实现农村广播电视户户通。积极组织开展节日文化、广场文化、社区文化、文化惠民演出、全民阅读等活动，不断满足群众日益增长的精神文化需求。

创新社会治理。严格落实领导干部接访责任，完善法律援助制度，依法规范信访秩序，充分运用法治思维、法治手段预防和化解社会矛盾。全面贯彻执行新《安全生产法》，建立健全隐患排查、安全防控体系和基层安全生产监管体系，进一步夯实企业主体责任，坚决遏制重特大事故发生。建立完善食药品监管体系，加强市县食药品监测机构建设，保障全市人民饮食用药安全。深化“诚信榆林”建设，建立全市社会信用体系的基本框架和运行惩戒机制。推广“金阳社区网格化管理模式”，建立规范的社区工作标准和评价机制。切实做好民间借贷和非法集资问题的化解处置工作。将社会稳定风险评估作为“前置程序”和“刚性门槛”，切实提高行政决策的科学性。全面推广城区巡防机制，加强重点区域、重点场所的综合治理，严密防范、依法打击各类违法犯罪和恐怖活动。启动安康医院建设，着力解决残疾人和重症患者吸贩毒问题。强化应急预案的针对性和实效性，不断提升应急管理工作水平。

以法治政府建设为核心全面加强政府自身建设。 深入贯彻落实党的十八届四中全会精神，加快建设职能科学、权责法定、执法严明、公开公正、廉洁高效、守法诚信的法治政府，为各项工作开展提供强有力的保障。

严格依法履行政府职能。坚持法定职责必须为、法无授权不可为原则，厘清权力边界，加快推进机构、职能、权限、程序、责任法定化，建立权力清单、负面清单和责任清单，及时公布行政许可、行政处罚、行政强制、行政征收等事项，坚决杜绝法外设权。在继续简政放权的同时强化事中事后监管，更加重视履行市场监管、社会管理、公共服务和环境保护等职能，切实解决好与群众直接相关的食品安全、市场管理、环境污染等问题。

努力健全依法决策机制。把公众参与、专家论证、风险评估、合法性审查、集体讨论决定作为重大决策的法定程序，进一步健全政府法律顾问制度，切实加强新型智库建设，积极推行网上审批，建立重大决策终身责任追究制度及责任倒查机制，确保决策制度科学、程序正当、过程公开、责任明确。加强对行政决策执行情况的考核评估，确保决策落实到位、执行到位。

切实规范行政执法行为。加快推进综合行政执法体制改革，积极探索和推行跨部门综合执法，着力解决权责交叉、多头执法问题。建立重大执法决定合法性审核、执法全过程记录等制度，细化量化行政裁量标准，提高执法规范化水平。落实行政执法责任制，加强执法监督，坚决查处以言代法、以权压法、徇私枉法等行为。严格执行首问负责、按期办结、督查考核制度，大力倡导敢于担当、埋头苦干、务实高效的工作作风。

不断强化对行政权力的监督和制约。自觉接受人大法律监督、政协民主监督和社会舆论监督，及时回应建议、意见和批评。加强政府内部权力制约，对政府资金分配使用等权力集中的部门、岗位，实行分事行权、分岗设权、分级授权、定期轮岗制度。全面推进政务公开，高度重视网络舆情，广泛开展公民进政府活动。深入推进市县政务服务中心标准化建设，加快市县乡三级政务服务体系互联互通，推行干部代办制度。落实党风廉政建设主体责任，强化行政效能建设，坚决制止不作为、乱作为、有令不行、有禁不止、推诿扯皮等不良行为。按照守纪律、讲规矩要求，深入开展“三严三实”教育活动，严格落实“八项规定”，严控“三公”经费支出，严肃查处各类违纪违法行为，以实际行动赢得全市人民的支持和信任。

各位代表！榆林转型发展正处于逆水行舟、不进则退的攻坚时期，唯有敢于担当、奋力前行，方能不辱使命、创造辉煌。让我们紧密团结在以习近平同志为总书记的党中央周围，在市委的坚强领导下，用实打实的作风干工作，以钉钉子的精神抓落实，为早日全面建成小康社会而努力奋斗！
